# Regulation of Ion Gradients across Myocardial Ischemic Border Zones: A Biophysical Modelling Analysis

**DOI:** 10.1371/journal.pone.0060323

**Published:** 2013-04-05

**Authors:** Steven Niederer

**Affiliations:** Biomedical Engineering, King's College London, London, United Kingdom; Georgia State University, United States of America

## Abstract

The myocardial ischemic border zone is associated with the initiation and sustenance of arrhythmias. The profile of ionic concentrations across the border zone play a significant role in determining cellular electrophysiology and conductivity, yet their spatial-temporal evolution and regulation are not well understood. To investigate the changes in ion concentrations that regulate cellular electrophysiology, a mathematical model of ion movement in the intra and extracellular space in the presence of ionic, potential and material property heterogeneities was developed. The model simulates the spatial and temporal evolution of concentrations of potassium, sodium, chloride, calcium, hydrogen and bicarbonate ions and carbon dioxide across an ischemic border zone. Ischemia was simulated by sodium-potassium pump inhibition, potassium channel activation and respiratory and metabolic acidosis. The model predicted significant disparities in the width of the border zone for each ionic species, with intracellular sodium and extracellular potassium having discordant gradients, facilitating multiple gradients in cellular properties across the border zone. Extracellular potassium was found to have the largest border zone and this was attributed to the voltage dependence of the potassium channels. The model also predicted the efflux of 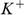 from the ischemic region due to electrogenic drift and diffusion within the intra and extracellular space, respectively, which contributed to 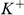 depletion in the ischemic region.

## Introduction

Myocardial ischemia is caused by reduced perfusion to regions of the heart leading to a localised reduction in supply of metabolites, limited waste removal and compromised ionic homeostasis. The first 10 minutes of ischemia are associated with an increased risk of arrhythmias peaking after 5–6 minutes [1]. During this period arrhythmias are commonly initiated within the border zone (BZ) separating viable, well perfused, tissue and the ischemic, underperfused, region [2]–[4]. Ischemia causes an increase in extracellular potassium (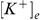), intra and extracellular proton concentrations (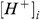 and 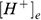, respectively), intracellular sodium (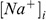) and intracellular calcium (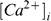) concentrations [5]. The dominant mechanisms for these changes have been attributed to a shift in the ATP/ADP ratio, which inhibits the Sodium-Potassium ATPase pump (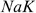) and increases the conductance of ATP-inactivated 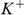 channels; respiratory acidosis causing an increase in 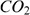; and metabolic acidosis, where a shift towards anaerobic respiration increases the production of 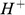 in the cell [5]. Inherently, these changes in ionic concentrations in the ischemic region lead to gradients in properties across the BZ, creating electrophysiological heterogeneities that are thought to favour the occurrence of arrhythmias [6]–[8].

Experimentally, the development of gradients of extracellular pH (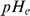) and 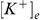 [9], [10] have been well characterised using ion sensitive electrodes. Intracellular metabolite gradients have been characterised by fluorescent NADH [11] and biopsy [12] measurements. However, less is known on the gradients of intracellular ions, in particular 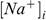, 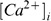 and 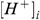, nor are the mechanisms that underpin the spatial and temporal evolution of these ion concentration gradients well characterised or understood. This study aims to investigate the spatial-temporal evolution of ion gradients across ischemic BZ and the primary regulators of the BZ size and rate of development.

Previous measurements of ion concentrations and metabolites across the BZ have either been performed at multiple locations but at a limited number of time points [10], [12] or have tracked the time evolution of ion concentrations but only from a limited number of locations [13], [14]. Furthermore, these measurements have only been able to characterise a subset of ions of interest across the BZ. The need to track the evolution of multiple ionic species in space and time to understand the gradients of cellular electrophysiology across the BZ motivates the use of biophysical computational modelling. Previous models of electrophysiology during acute regional ischemia have simulated the effects of these spatial gradients but have not simulated their time evolution [15]–[17]. More recent work has simulated the time evolution of 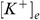 gradients [18], but have not considered other ionic gradients, the effects of nonlinear interactions between 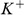 and other ions, the effect of 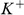 diffusion in the intracellular space or the effects of potential gradients on ion diffusion.

In this study a new model of cardiac tissue electrophysiology is developed to investigate the spatial-temporal evolution of ionic concentrations across the ischemic BZ, during the first 5 minutes of reduced perfusion. The proposed model extends the conventional bidomain equations to explicitly link membrane potential to ionic concentrations and enforces ionic species conservation. A model of ion regulation across the cell membrane is then developed, parameterized, validated and coupled to the tissue model. This combined model is then used to investigate the spatial and temporal dispersion of ions across the BZ.

## Methods

To model the evolution of ionic concentrations in the presence of multiple ionic gradients, electric gradients and heterogeneous tissue properties requires the development of a new set of equations for modelling the myocardium. In the next section the equations to model the BZ are derived and a model of passive cell membrane ion regulation is developed and validated. The changes to the cell membrane model to simulate 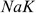 inhibition, 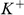 channel activation, respiratory acidosis and metabolic acidosis are then described.

### Tissue Model Derivation

Consistent with previous models of cardiac tissue electrophysiology the myocardium is represented as a two phase medium, with each point in the domain containing a fraction of intra and extracellular space. This gives rise to the scaling variables.
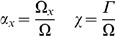
(1)where 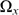 is the volume of the 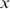 space in a unit of 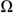 myocardium volume, 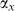 is the volume fraction of space 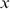 (with 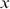 corresponding to the extra (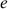) or intra (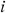) cellular space), 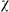 is the interspace surface area per unit volume myocardium and 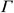 is area of the interspace surface. The intracellular volume fraction (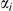) can be separated into sub volume fractions representing the cytosol (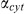), mitochondria (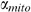) or the sarcoplasmic reticulum (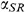) to model distinct subcellular spaces, as described below. At each point in space there exists an intracellular potential (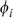), an extracellular potential (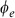), a transmembrane potential (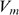) and an intra and extracellular ion concentration for each of the ion species in the model. The movement of each ion species in each domain is driven by diffusion, due to a gradient in ion concentration, and drift, due to a gradient in the electric field. This movement is described by the Nernst-Plank equations
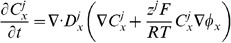
(2)where 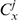 is the concentration of unbound ion 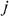 in space 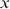, 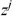 is the charge of ion 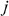, 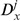 is the effective diffusion of ion 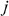 in space 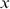, 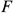 is Faraday's constant, 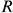 is the gas constant, 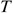 is the absolute temperature and 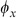 is the potential in the space 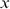. The conventional Nernst-Plank equations are adapted to represent the movement of ions across the cell membrane between the intra and extracellular spaces. This gives
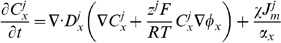
(3)where 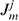 is the flux of ion 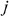 across the cell membrane. 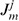 is defined in units of mM ms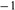 mm. The surface separating the two regions is modelled as a simple capacitor and defining the transmembrane potential to scale with intracellular charge gives
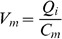
(4)where 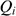 is the intracellular charge per unit cell membrane area and 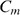 is the membrane capacitance per unit cell membrane area. The charge on either side of the membrane is assumed to be equal but opposite, giving



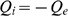
(5)Separating the concentration of each ion species in the intra and extracellular space into ions that are membrane bound and make up the membrane charge (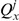) and those that are in solution gives.
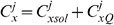
(6)where 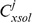 and 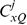 are the ions in solution and membrane bound ions, respectively. By assuming charge neutrality for the ions within the solute gives:



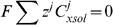
(7)Multiplying Eqn 6 by the ion species charge and Faraday's constant, then summing over all ion species in space 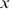 gives.
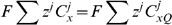
(8)


Converting the concentration of ions per unit volume in domain 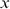 to charge per unit cell membrane area and introducing a static charge term (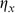) that characterises all charge not attributable to 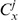, gives
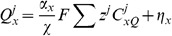
(9)


Combining with Eqn 8 gives
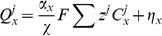
(10)


This defines the charge on the membrane as equal to the unbalanced charge in space 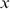. Using the charge balance Eqn 5, gives
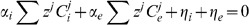
(11)


Differentiating Eqn 11 with respect to time, substituting in Eqn 3 and recognising that all transmembrane fluxes are balanced, provides
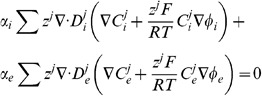
(12)this ensures that there is no net charge accumulation in any unit volume of myocardium. Defining the relationship between the intra and extracellular potentials gives



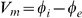
(13)Using Eqn 4 and the definition of charge (Eqn 10), then gives the algebraic definition of the transmembrane potential:
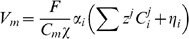
(14)


Rearranging Eqn 13 and substituting in the definition of 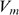 from Eqn 14 allows 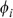 to be defined in terms of 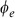 and 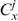. Combining this definition of 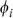 with Eqns 14, 13, 12, 3 and 2 then represents a closed set of equations. These equations are equivalent to the bidomain equations in the case of a single charge carrier and no gradient in ion concentrations, as shown below.

In cardiac myocytes many important ions, including 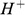 and 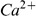 are heavily buffered, both within the cell and the extracellular space. To account for buffering the free and buffer bound fraction of 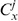 are calculated. In general, as 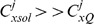 the effect of ions bound to the cell membrane will not be included in the buffering equations for simplicity. This gives
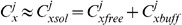
(15)where 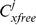 are the unbound ions and 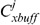 are the ions bound to buffers. At this time all buffers will be treated as rapid and to a single representative buffer species, giving
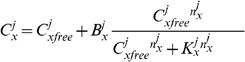
(16)where 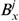, 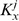 and 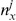 are the concentration, binding affinity and Hill coefficient, respectively, for the buffer of ion 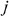 in space 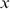. Assuming that ions bound to buffers are immobile, only 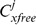 is used to calculate the diffusion and drift of ions in Eqn 3, and similarly in Eqn 12. As binding of ions to a buffer implicitly removes a charged binding site located on a static protein, Eqn 14 remains unchanged.

Due to the complex anatomy of the cardiac myocyte many sub volumes exist within the cell that affect ionic concentrations. The sum of the volume fraction (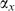) values must be less than, but do not have to be equal to, one, allowing the model to represent any volume fractions that are not directly accessible by ions. In particular the 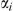 variable can represent all space in the cell or can be substituted for 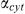 representing the volume fraction of the cytosol (a sub volume of 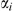). This allows the effects of SR, mitochondrial or other subcellular structure volumes on intracellular ionic concentrations to be accounted for in the model.

#### Equation summary

The modelled equations are given by
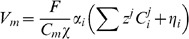
(17)

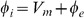


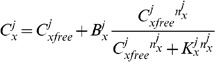


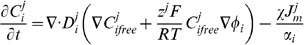


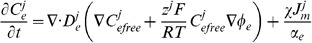


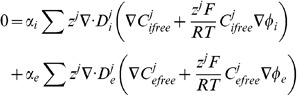
where it is important to note that for non buffered ions 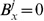 and 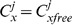.

#### Consistency with bidomain equations

Imposing the implicit assumptions of the bidomain equations that charge carriers are not buffered, ion concentrations are homogenous and charge is carried by a single carrier to Eqn 18, the bidomain equations can be derived. Assuming homogenous ion concentrations and considering the case of the intracellular space reduces Eqn 18 to
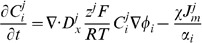
(18)


Differentiating Eqn 14 for the intracellular space gives
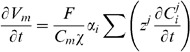
(19)substituting Eqn 18 into Eqn 19 gives



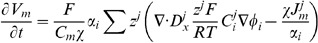
(20)As ion concentrations are homogenous conductivity is defined as
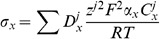
(21)


Then Eqn 20 reduces to
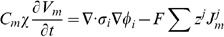
(22)


Applying the single charge carrier assumption and converting from ionic flux to current gives the first bidomain equation
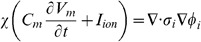
(23)


The second bidomain equation is readily derived from applying the homogenous ion concentration assumption to Eqn 12 and multiplying by Faraday's constant (to convert from conserving ion flux to current) giving
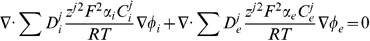
(24)


Substituting in the definition of conductivity from Eqn 21 then gives the second bidomain equation
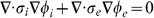
(25)


### Modelling the Membrane Fluxes

Cardiac electrophysiology is predominantly determined by the movement of 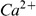, 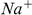 and 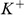. For charge neutrality 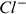 must also be included in the model. To simulate the evolution of acidosis requires the inclusion of 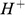, 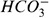 and 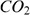 in the model. All of these ions (and 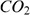) were modelled in the intra and extracellular space, with 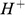 and 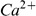 being buffered. The goal of the model, in this study, was not to track the propagation of the action potential but to simulate the gradients of ions that exist over the BZ. These ion gradients were modelled based on the diastolic properties of the cell. This assumption was also a requirement to enable the simulation of minutes, while remaining computationally tractable.

The membrane ion transport pathways are described first for 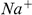, 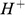 and 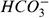. Ion specific channels are then described that balance the flux of each ion species. The channel and transporter densities were determined by imposing zero net flux for each ion species, using the relative densities of 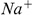 and 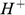 transporters recorded experimentally, and intra and extracellular ionic concentrations and membrane potential values derived from the literature. Where possible experimental data was taken preferentially from rabbit or guinea pig data at body temperature. This limited number of constraints then allowed the model transporters and channel densities to be uniquely determined.

#### Sodium regulation

The model of 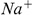 regulation included representations of the 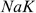, sodium calcium exchanger (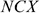), sodium hydrogen exchanger (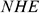), sodium bicarbonate co-transporter (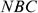) and a lumped sodium channel (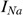). 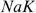 was modelled using the thermodynamically consistent equation set proposed by Smith and Crampin [19]. This model was subsequently revised by Terkildsen et al., [20] and this parameter set that was used here. The model for 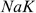 was fitted to guinea pig data as limited rabbit data was available. However, the maximum flux was rescaled to match rabbit data, as described below.

The 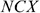 model was taken from Weber et al., [21]. The model has been fitted to rabbit experimental data at 37°C. The 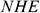 model was based on the model developed by Crampin and Smith [22] and reparameterized by Niederer and Smith [23]. In this study extracellular 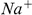 and 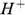 regulation of 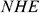 were included. This model was fitted predominantly to sheep Purkinje data [24], although the 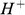 dependence of 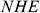 remains relatively consistent between species [25]. The 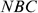 model was taken from Crampin and Smith [22]. The model assumes 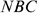 is electro neutral, which is true for only part of the 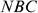 population [25]. There was not sufficient data to fully characterise the electrogenic and electro neutral forms of 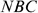, hence the electro neutral model was used. Background 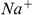 flux across residual open fast 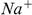 and persistent 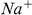 channels is limited when the cell is quiescent. However, some flux is still present [26] and a simple lumped background ionic flux equation was used, given by
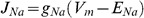
(26)to model the residual 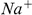 flux across any open 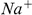 channels. The same equation form was used for modelling all background ion channels.

#### Proton regulation

In the proposed model 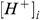 was regulated by 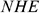, described above, chloride-hydroxide exchanger (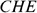), hydrolysis and buffering. Background 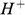 leak or other 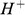 exchangers were not considered in the general model of 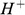 regulation, described here, but do include models of 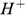-lactate exchange and intracellular metabolism derived 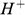 sources in the model of ischemia, described below. The 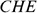 model comes from Niederer et al., [27] and was fitted to guinea pig data at 37
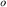
C. The hydrolysis of 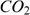 into 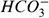 and 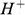 was governed by
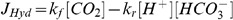
(27)where 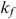 and 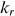 are the forward and reverse rates of hydrolysis. Hydrolysis occurs in both the intra and extracellular space and the rate constants were assumed to be the same in both domains. 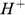 buffering in the intra and extracellular space is due to mobile and static 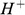 buffers and 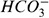 [28]. The intra and extracellular buffering of 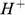 were assumed to be instantaneous and represented by a single population of buffers. To reduce the model size, partial differential equations were only solved for the total concentration of 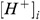 or 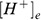. The concentration of free ions were then calculated by
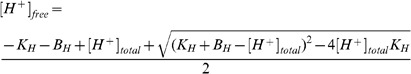
(28)where 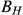 and 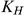 represent the concentration of the buffer and the binding affinity, respectively. This buffer model was used for both intra and extracellular 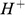, with a separate set of parameters for each ion.

#### Calcium regulation

A simplified model of intracellular cardiac 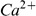 was developed assuming that 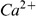 in the intracellular space reaches an approximate equilibrium over the time scales of interest. Furthermore, SERCA ATPase function was modelled with a Hill coefficient of one as opposed to two, to allow the definition of 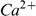 to remain deterministic. The intracellular space was assumed to consist of a sarcoplasmic reticulum (SR) and a cytosolic space. The subsarcolemmal and dyadic space are small and are also likely to be in equilibrium with the cytosolic 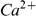, so were not included in the model. The 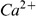 dynamics were described by
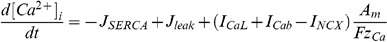
(29)

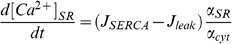
(30)

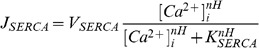
(31)

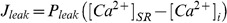
(32)

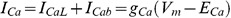
(33)where 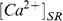 is the SR 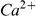, 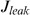 is the flux of calcium out of the SR, 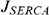 is the uptake of 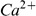 by SERCA, 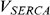 is the maximum SERCA flux, 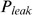 is the diffusion permeability of the SR membrane, 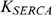 is the binding coefficient of 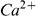 to SERCA, 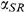 and 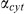 are the volume fractions of the SR and cytosol, respectively, 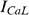 is the L-type calcium channel and 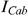 is the background 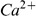 channel. In this model the background and L-type 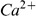 channels were modelled as a single lumped generic 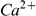 channel. Introducing cytosolic buffering, ignoring the effects of SR buffering and setting 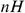 as one and assuming that the cytosol and the SR are in equilibrium then gives



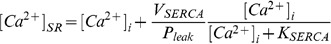
(34)

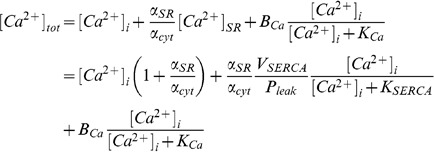
defining



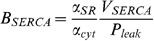
(35)

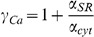
(36)and collecting terms gives



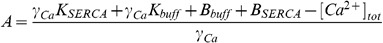
(37)

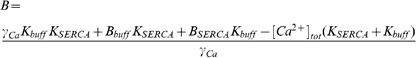
(38)

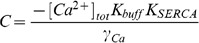
(39)

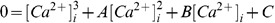
(40)


As 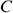 is always negative the cubic always has at least one positive real root for possible values of 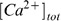. The value of 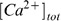 was then found using the root finding method first proposed by Francois Viete in 1600 and reused more recently by Faber and Rudy [29]:
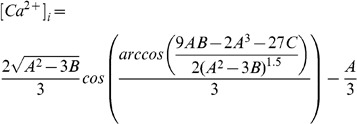
(41)


The model of 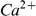 dynamics assumes that all 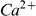 buffers were static and that the transport of ions via mobile buffers was accounted for in the effective diffusion parameters of free 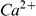. It is possible to extend the proposed model to include mobile buffers but they were assumed to play a secondary role in the current model. 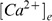 was assumed to be buffered by a single species and was modelled using the same framework described above for 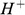 (Eqn 28).

#### Chloride regulation

In this model 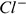 homeostasis was maintained by the 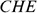 and the 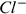-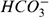 exchanger (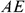), which bring 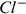 into the cell, and a 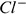 channel that allows 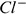 to flow out of the cell. The 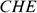 model is described above. The 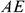 model was taken from Crampin and Smith [22] and was developed using guinea pig data at 37
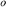
C. The 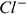 channel uses the conventional background channel formulation. A linear 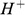 dependence of the background 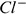 current was added to the model based on observations from Komukai et al., [30].

#### Potassium regulation

In dynamic action potential models of cardiac electrophysiology there are a large number of 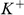 channels [31] that bring 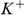 into the cell. This influx was balanced by the 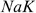 pump, described above, that extrudes 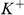. For the passive membrane model, all of the 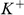 channels were lumped into a single background current (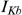) formulation that was set to balance the flux of 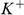 on 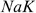. It was assumed that the membrane potential and 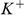 reversal potential are the dominant factors affecting this channel and other forms of regulation have not been considered.

#### Bicarbonate regulation


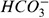
 was assumed to be regulated principally by hydrolysis and through 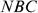 and 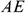. All of these components have been described above and it was assumed that there are no other 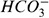 pathways across the membrane.

#### Carbon Dioxide


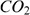
 is regulated primarily through hydrolysis and can diffuse relatively freely across the membrane. The model of hydrolysis is described above and 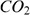 diffusion was assumed to obey Fick's law.

### Model Parameters

For each transmembrane ion pathway described above, all kinetic, binding affinity and membrane potential dependencies were taken from the original models. Here the definition of geometrical parameters, ionic concentrations, buffering parameters and the density/scaling of each transmembrane pathway are motivated from data in the literature.

#### Geometrical parameters

The extracellular space is estimated to be between 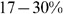 [32]–[35], 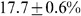 and 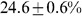 [33], [36] of the volume of the heart in rabbit, cat, and rat hearts, respectively. This gave an 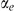 value of 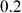 leading to an 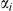 value of 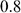. The surface to volume ratio of a cell is reported as 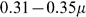 m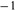 [37], [38] in rat and rabbit myocytes, corresponding to a 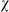 value of 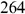
mm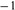. The relative SR volume was set to 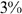 of intracellular volume, giving an 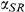 value of 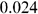, based on reported values of 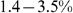 of cell volume [37], [39], [40] in mouse, rat and swine. The relative mitochondrial volume (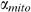) was set to 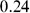 based on an estimated mitochondrial cell volume fraction of 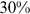 [37], [40]. The cytosol volume fraction was set to 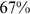 of the intracellular space, resulting in an 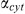 value of 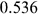. To account for the effects of subscellular domains on intracellular ionic concentrations in the model, all references to 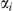 in Eqn 18 were replaced by 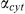. A summary of geometrical parameters is given in Table 1.

**Table 1:** Geometric variables.

Variable	Value
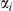	0.8
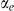	0.2
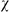	264 mm 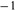
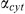	0.536
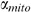	0.24
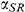	0.024

#### Intracellular ionic concentrations


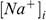
 has been measured using SBF1 fluorescence and 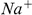 sensitive electrodes. A significant range of values have been reported from 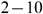
mM [26], [41]–[44] to 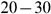
mM [34], [45], [46]. Early measurements of intracellular ionic concentrations were performed using ion sensitive electrodes. These experiments measure ion activity and not ionic concentration and are often performed in multi-cellular preparations, confounding measurements. For these reasons 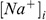 in quiescent myocytes was set to 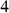
mM, consistent with recent calibrated fluorescent measurements in isolated rabbit myocytes [26], [44].

No fluorescence dye is routinely used for measuring 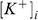. Using ion sensitive electrodes Lee et al., [45] were able to calibrate their measurements of ion activity in rabbit myocytes using an estimated 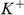 activity coefficient of 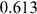, giving a value of 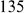
mM. This compares with a range of 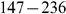
mM calculated by applying the Lee et al., 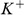 ion activity coefficient to ion activity measurements in rabbit, cat and guinea pig [34], [43], [47], [48]. Alternate measurement using flame emission spectrometry by Powell et al., [49] measured 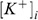 in rat myocytes, giving a concentration of 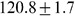 mM. Given the lower values of the two calibrated measurements, 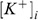 was set to 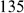 mM.

No dye is routinely used for measuring 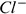 concentration in cardiac cells, however, 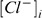 can be measured using ion sensitive electrodes. 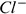 activity has been reported as 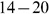 mM [35], [48], [50], [51] in sheep, rabbit and guinea pig heart cells. Estimations of 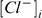 from total tissue 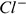 concentrations have resulted in values of 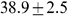 mM [35] and 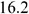 mM [34] in rabbit cells and 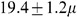 mol/g dry wt (or 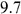 mM using the 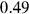 mM per 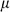
mol/g dry wt scaling factor from Bers [52]) in rat. The higher value of 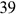 mM may be attributed to the higher extracellular space used in these calculations (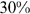 as compared to 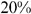). Considering the relative convergence of values 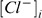 was set to 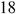 mM.

Resting free 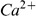 is measured using calibrated fluorescence measurements. These measurements range from 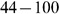 nM in rabbit and guinea pig preparations [53]–[57]. Given this consistency the 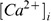 will be set to 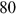 nM. SR 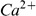 concentration is calculated from integrating the current across the cell membrane following the release of 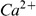 from the SR in response to caffeine. These measurements show two populations with high values in rat (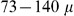
M [58]–[61]), canine (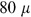
M [62]), rabbit (87–106 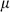
M [60], [61]) and ferret (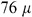
M [63]), compared to lower measurements in guinea pig (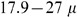
M [58], [64]). Given that the majority of species have a higher reported concentration, including rabbit, simulations were run with SR 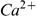 load set to 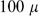
M. The buffering of 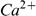 can be described by Hill equation(s), mass action equation(s) or a constant buffering power. To compromise between biophysics and complexity, buffering was modelled by a single Hill equation. Hove-Madsen and Bers [65] fitted 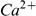 buffering in rabbit myocytes using two Hill curves; however, the lower affinity buffer will not play a significant role at passive diastolic 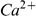 concentrations. For this reason the high affinity site, with cooperativity reduced from 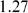 to unity, was used to model 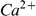 buffering, giving a buffer concentration of 208.98 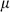
M (converted using a scaling factor of 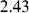 from Bers [52]) and an affinity of 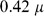
M. This model of 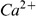 buffering in rabbit is similar to the concentration/affinity values of 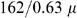
M [66], 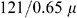
M [67] and 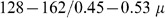
M [68] values measured in other species.


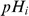
 has been measured using 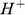 sensitive electrodes and fluorescence dyes. Measurement of 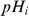 consistently falls within the range of 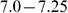 [57], [69]–[71] in either HEPES or 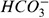 buffered solutions. In the model 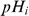 was set to 7.1. 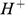 are heavily buffered in the cytosol by intrinsic buffers and 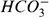. Here the buffering of 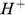 by 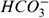 was modelled explicitly and the intrinsic buffers were assumed to be in rapid equilibrium. Leem et al., [70] measured (and modelled) 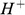 buffering by two populations of buffers with binding affinity pK values of 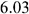 and 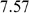 and concentrations of 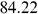
mM and 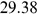
mM. Zanbioni et al., [25] differentiated between mobile and fixed buffers and found that the fixed buffers had a consistent concentration of 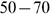
mM and binding affinity (pK) value of 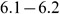 across rat, rabbit and guinea pig, while the mobile buffers had a constant 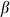 value of 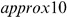. Fitting a single buffering curve to these two models over a pH range of 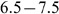 gives concentrations of 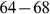
mM and pK values of 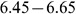, which gave a value of 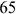
mM and pK value of 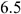 for this model.

#### Extracellular ionic concentrations

The concentration of the majority of ions in the extracellular space have been measured in canine [46], [72], rat [36], [73] and cat [74] hearts. These measurements provide a consistent range of ion concentrations for 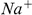, 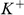 and 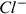, giving 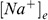 as 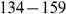
mM, 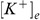 as 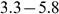
mM and 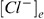 as 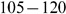
mM. In the model 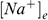 was set to 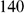
mM, 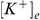 was set to 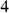
mM, consistent with measurements in guinea pig hearts [75] and rabbit atrium [76] and 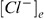 was set to 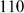
mM. The 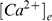 values reported range from 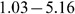
mM, however, these values do not differentiate between buffered and ionized 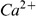. The properties of 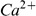 buffering in the extracellular space are not well characterised and are generally ignored in previous cardiac cell models. To approximate the buffering properties of extracellular 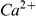 using a simple single species steady state mass action model, with no cooperative binding, requires two parameters, the concentration of the buffer and the binding affinity. Assuming the ratio of free 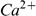 in the extracellular space to bound ions is similar to serum [77] and assuming that the primary buffer of 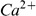 in the extracellular space are phospholipids, then extracellular 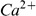 buffering will have a binding affinity of 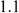
mM [78], within the range observed across multiple species [79], and a buffer concentration of 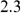
mM based on a free 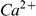 concentration of 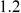
mM and assuming 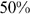 of 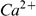 are bound to buffers [77]. 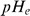 was set to 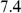, to be consistent with the majority of experimental studies [57], [69]–[71] and measurements across a range of species [80]. Limited measurements were available to model 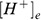 buffering, however, Yan and Kleber [81] reported 39 mM of buffered 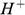 in the extracellular space. By assuming similar binding affinities for the intra and extracellular buffers and that 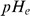 is 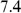, then gave a concentration of extracellular proton buffers of 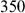
mM. A summary of ion concentrations and buffering parameters are given in Table 2 and Table 3, respectively.

**Table 2:** Intra and extracellular free ion concentrations.

Ion	Concentration (mM)	
	Intracellular	Extracellular
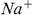	4.0	140
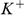	135	4.0
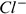	18	110
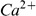	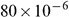	1.2
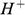	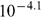	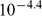
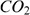	1.17	1.17

**Table 3:** Buffering Parameters.

Parameter	Value (mM)	
	Intracellular	Extracellular
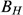	65	350
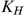	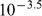	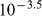
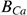	0.209	2.3
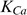	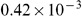	1.1

The concentration of 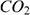 was calculated using the parameters proposed and measured for guinea pig ventricular myocytes at 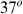
C by Leem and Vaughan-Jones [82]. The concentration of 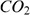 in the extracellular solution was calculated using
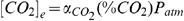
(42)where 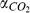 is the solubility of 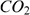, set to 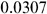 mM mmHg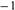 from human measurements at pH 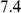 and 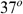
C [83], 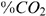 is the fraction of air that is 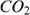, set to 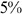 at baseline and 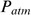 is atmospheric pressure, set to 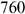 mmHg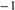. This gives a partial pressure of 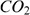 (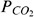) of 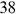 mmHg, consistent with although slightly higher than the 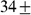
3 mmHg measured in rabbit hearts [84]. The hydration of 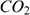 was modelled by a mass action reaction (Eqn 27). The 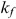 and 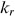 values were measured and modelled by Leem and Vaughan-Jones in guinea pig myocytes at 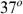
C [82], giving values of 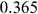
s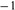 and 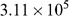
s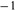, respectively, resulting in an equilibrium constant of 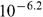. The rate of hydration of 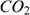 was assumed to be similar in both the intra and extracellular space. The movement of 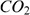 across the cell membrane was modelled by Fick's law. Previous models have used permittivity values of 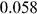
mms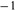 [82], based on measurements in red blood cells. This value was reused despite its lack of species and cell type consistency, as there were no recent studies characterising the permeability in cardiac myocytes and the high permeability leads 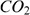 to be close to equilibrium between the intra and extracellular spaces.

#### Current, exchanger and pump densities

The model of each transmembrane ion pathway (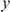) was separated into a kinetic regulatory component (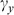) dependent on transmembrane potential and ionic concentrations, and a scalar (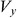) representing either the maximum flux or channel conduction of the pathway. The flux across a pathway (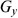) is then given by
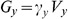
(43)


The 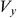 values were determind by calculating all of the 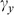 values (excluding 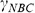, 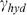 and 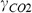 as 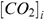 and 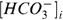 were unknown) using the ionic concentrations in Table 2 and assuming a membrane potential of −80 mV. The remaining unknown 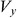 and ionic concentrations were then determined from a limited number of measurements and by enforcing a zero net flux condition described by
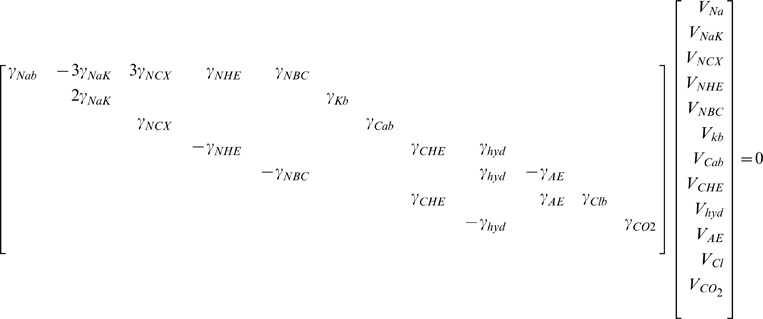
(44)


As the parameters for hydrolysis and permeability of the cell membrane to 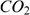 (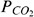, 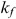 and 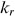) were derived from the literature, the constraints on 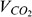 and 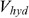 were used to determine the concentrations of 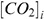 and 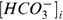, respectively. Measurements of transmembrane 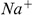 influx gave 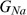 as 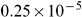 mM ms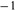, 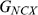 as 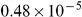 mM ms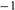, 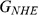 as 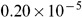 mM ms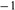 and 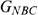 as 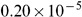 mM ms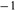 [85]. The 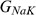 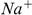 flux was assumed to be equal to the sum of all 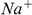 influx. As the model does not include 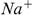-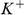-2-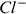 co-transporter (NaK2Cl) or 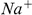-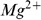 exchanger (NaMg), primarily due to the limited data to constrain the kinetics, 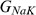 was reduced from the flux measured by Despa et al., [85] to 1.13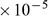 mM ms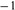. Setting these 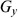 values defines 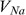, 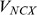, 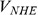, 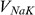, 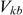 and 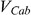. In the experimental and modelling work by the Vaughan-Jones group [70], [86] the relative size of 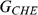 to 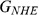 is 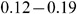 at 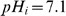, this gave an estimated ratio of 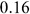. Scaling 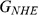 determined from [85] by 0.16 provided an estimate of 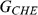 of 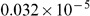 mM ms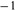. This allows 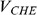 and 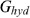 to be defined. 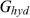 must be balanced by 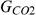 and as 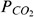 and 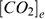 were known, this flux was used to set 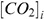. Combining the 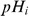 and 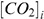 concentrations with the defined hydrolysis parameters and the known value of 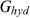 then gave 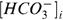. Knowing 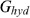 and 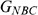 gave 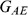 and hence 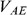. Similarly, 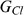 was calculated from 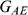 and 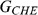,which gave 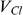. Finally, 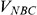 was calculated using 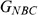 and 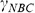, calculated using the derived 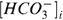 value. By automating this parameter derivation process the model parameters could be updated to ensure static ion concentrations, and hence membrane potential, for any perturbation in model parameters or ionic concentrations. Parameters for all simulations in this study were derived following this process.

#### Intracellular calcium dynamics

The intracellular regulation of 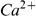 was treated as an equilibrium system. This resulted in the SR effectively acting as an additional buffer on 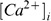. The parameters 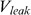, 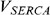, 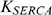, 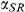 and 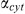 were defined from enforcing a zero net flux constraint and experimental measurements. Measurements of 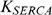 are similar in rat and rabbit [65] and have been reported as 0.260–0.350
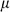
M in rabbit [65], [87] and 250–280
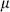
M in rat [65], [87], [88]. A value of 0.3
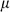
M was used in the model. 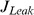 is reported as 0.001–0.012 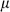
Mms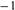 in rabbit, mouse and rat [54], [89]–[91]. A value of 0.01
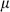
Mms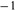 was used in the model. Enforcing a zero net flux constraint on the SR gave 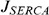 as 0.038 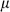
Mms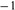, comparable with values of 0.03–0.08
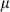
Mms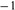 measured in rabbit [65], [87], [92] with units converted using scale factors from Bers [52].

### Membrane Model Validation

**Figure 1:**
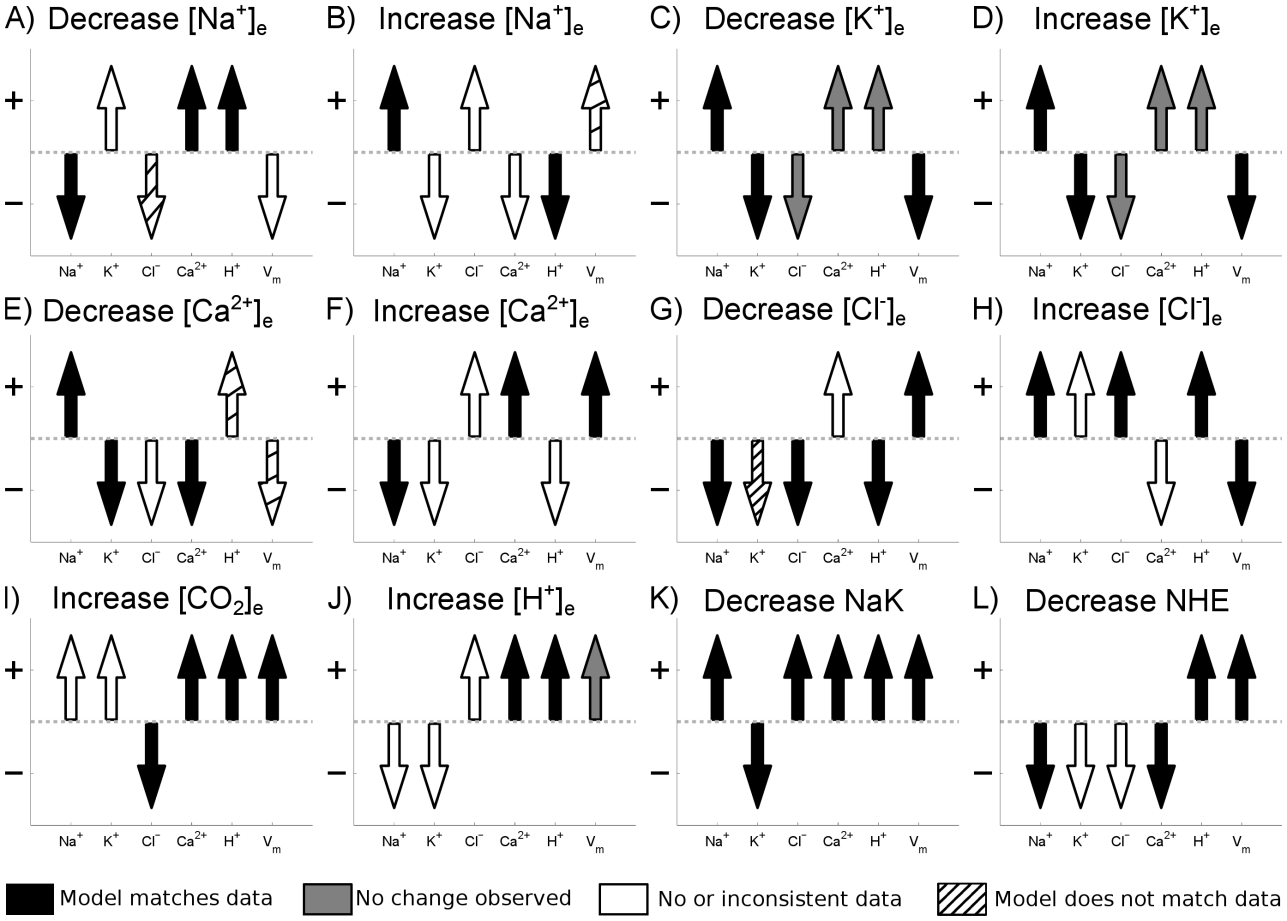
Cell membrane model validation. Arrow direction shows model prediction for the change in intracellular concentrations and membrane potential for a change in extracellular ion concentrations or inhibition of membrane transporter as indicated by the panel label. The color of the arrow indicates how the model compares with experimental data, summarised in Table 4. Black arrows indicate where the model matches experimental data, white arrows indicate where there is no or inconsistent data, gray arrows indicate where no change was observed experimentally and striped arrows indicate where the model does not match experimental data. Data was considered inconsistent if different studies reported opposite changes in intracellular ion concentration or membrane potential in response to a change in extracellular ionic concentrations (for example the change in membrane potential following a decrease in 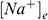). In cases where one study reported no change in intracellular ion concentration or membrane potential and another found a change, it was assumed that the change was correct (for example the change in 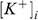 in response to an increase or a decrease in 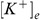). For example in panel A) corresponding to a decrease in 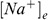 (as indicated by the panel label), the experimental data comes from row 1 of Table? 4. The model predicts that 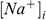 decreases and that 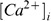 and 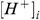 increase, consistent with experimental measurements and the arrows are shaded black. There is no consistent observed change in the transmembrane potential so the 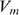 arrow remains white. No data is available for the change in 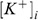 in response to a decrease in 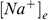 so the 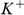 arrow remains white. The model predicts a decrease in 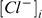 yet experimental measurements found an increase, hence the 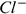 arrow is striped.

To validate the passive membrane model, a series of tests based on the response of the cell models intracellular ionic concentrations and transmembrane potential to changes in extracellular ionic concentrations or inhibition of major ion transporters was performed. In order to maximise the number of tests the model was compared against data from cardiac cells, regardless of temperature, species or preparation type. This maximised the number of tests but meant that a quantitative comparison was not valid and so only a qualitative comparison was performed. A summary of experimental data used in the validation is provided in Table 4. Fig. 1 shows the comparison between the model and data. From the 72 simulations performed 43 matched the experimental data, data was not available or was inconsistent for 19, changes were too small to be measured in 5 and the model did not match experiments in 5 cases.

**Table 4:** Qualitative changes in intracellular ionic concentrations or membrane potential in response to changes in extracellular ionic concentration or inhibition of membrane transporters.

Protocol		Change in Concentration or Potential						Reference
Variable	Change	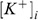	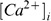	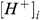	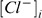	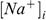	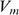	
1-24-9 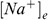								
	Decrease		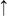	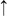	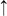	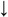	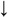 x2 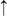 x4	[160]–[163]
								[164]–[166]
	Increase			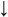 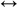		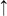	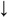	[167], [168]
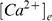								
	Decrease	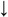	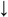	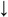		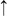	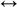 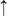	[163], [169], [170]
	Increase		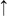			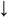	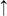	[171], [172]
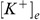								
	Decrease	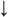 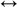	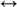	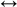	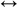	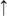	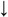	[160], [161], [173], [174]
								[71], [162], [175], [176]
	Increase	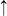 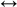	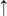 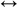	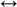	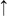 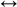	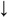	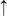	[50], [161], [175], [177], [178]
								[131], [173], [176], [179], [180]
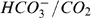								
	Increase		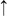	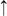	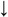		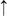	[71], [166], [181]–[183]
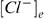								
	Decrease	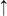		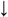	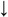	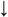	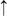	[184]–[186]
								[50], [71], [187]
	Increase			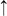	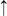	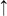	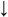	[50], [184], [187]
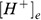								
	Increase		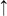	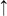		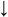 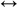 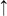	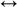	[161], [165], [166], [188]
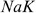								
	Inhibition	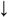	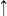	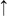	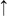	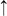	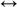 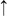	[43], [170], [175]
								[35], [160], [177], [189]
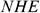								
	Inhibition		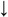	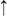		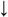	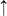	[44], [166]

### Diffusion Parameters

The tissue model required the definition of diffusion parameters for each of the ionic species in the intra and extracellular space. The diffusion parameters used in the model were all for the apparent diffusivity of free ions, lumping the effects of any mobile buffers, tortuosity and gap junctions into a single diffusion parameter. Using Eqn 21 the conductivity parameters were shown to be directly related to the conductivity parameters in the bidomain equations. From the review of bidomain conductivities and relative values in the intra and extracellular space by Roth [93] a conductivity value of 0.25 Sm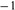 was taken for both the intra and extracellular space. This conductivity, along with the ionic concentrations from Table 2, were used to derive the diffusion parameters. The apparent diffusion of 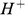 and 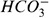 are 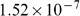
mm
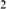
ms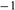 and 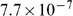
mm
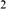
ms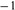 in the >intracellular space [86] and were assumed to be similar in the extracellular space. The apparent diffusion constant of 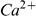 (including the effects of mobile buffers) has been estimated from experimental measurements in previous modelling studies as 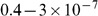
mm
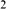
ms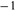 [94]–[96]. Here a value of 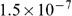
mm
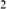
ms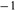 [94] was used and the effect of diffusion in the SR was not included, as it was assumed to be non contiguous between cells. It was assumed that 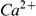 diffusion is limited by buffering, resulting in the same value in the intra and extracellular space. 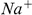, 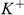 and 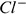 were all assumed to have a common diffusion coefficient in either the intra or extracellular space as these ions are only nominally buffered and are only affected by gap junctions and tortuosity. Diffusion of 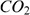 was estimated from previous modelling/experimental studies of 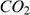 in tissue as 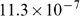
mm
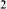
ms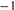 [97], and was assumed to be the same in the intra and extracellular space. Solving Eqn 21 for the diffusion of intra and extracellular 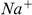, 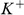 and 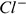 gives the diffusion parameters summarised in Table 5.

**Table 5:** Effective ion diffusion parameters.

Ion	Diffusion Constant (10 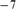 mm 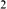 ms 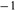 )	
	Intracellular	Extracellular
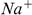	7.7	12.9
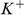	7.7	12.9
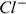	7.7	12.9
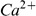	1.5	1.5
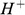	1.52	1.52
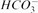	7.7	7.70
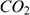	11.3	11.3

Finally, the effects of 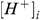 on gap junction conductivity were included. Measurements in cell pairs by Swietach et al., [98] have demonstrated that the permeability of gap junctions has a biphasic dependence on the 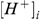 concentration. To introduce these effects into the model the intracellular diffusion constants were scaled by
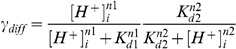
(45)where 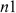/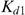 and 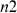/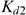 are the cooperativity and binding affinity for the activation and deactivation of gap junctions by protons, respectively and 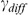 scales all intracellular diffusion constants. Using the measured parameters from end-to-end cell pairs in Swietach et al., [98] 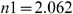, 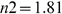, 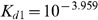
mM and 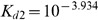
mM. In this model of proton effects on gap junction permeability there is an implicit assumption that the gap junction permeability plays a dominant role in defining diffusion. At this stage no other regulators of permeability were included in the model, notably 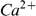 regulation is absent but this can readily be included in the modelling framework as required.

### Simulating Ischemia

In this study the effect of 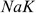 inhibition, 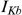 activation and respiratory and metabolic acidosis during ischemia on cell ionic homeostasis were considered. This list is not exhaustive and absent factors are discussed below. Ischemia was modelled by respiratory acidosis, metabolic acidosis, increased 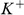 channel conductance and decreased 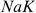 function. The specific time course of each of these changes is poorly characterised. Previous modelling studies have assumed that changes in cell function with ischemia have evolved linearly [99] or as a nonlinear function of prescribed metabolite concentrations [20]. To avoid any undue bias from the arbitrary selection of a time course, all changes are initially considered instantaneous.

NaK inhibition and 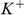 channel activation were modelled by scaling the respective fluxes. Respiratory acidosis was assumed to result from an imbalance of production and washout of 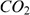. To simulate this, an (implicitly electro neutral) intracellular source of 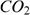 was introduced into the ischemic region. There was no flux of 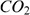 out of the extracellular space and this resulted in a build up of 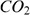 in both the intra and extracellular space in the ischemic region.

To model metabolic acidosis required the introduction of an additional intracellular 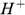 source. However, introducing a source of cations into the cell compromised the conservation of charge constraint. To provide an electro neutral source of 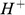 required the concurrent introduction of a source of anions that match the production of 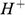 inside the cell. It was implicitly assumed that the anions entered the cell in an electro neutral form with an 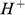 bound, and the anions and 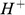 separate within the cell due to metabolic processes. The source of 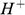 in ischemia is likely to be due to increased ATP production through glycolysis [100]; in the absence of oxygen this also results in increased lactate production. As lactate readily dissociates from 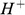 it was assumed that the anion source that matches 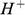 flux has similar characteristics to lactate including transmembrane regulation via the lactate-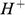 membrane exchanger MCT1 [101]. Given the ambiguity in structure, MCT1 was modelled as an ordered exchanger, using the kinetic parameters from Vinnakota and Beard [102] and the maximum influx value of 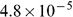
mMms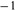 recorded in guinea pig myocytes [103]. In the absence of metabolic acidosis, lactate concentration was assumed to be nominal, consistent with the small flux of lactate observed in rabbit hearts under normal conditions [104]. Model simulations were performed on a 1D strand with length 32 mm, oriented in the preferred conduction or fibre direction, with a transition between ischemic and viable tissue at 16 mm to ensure the simulation captured the BZ width with nominal boundary condition artefacts. The transition between viable tissue and ischemic tissue was approximated by a Hill equation with a Hill coefficient set to ensure a steep transition between the ischemic region and viable tissue over 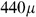
m, consistent with the rapid drop in oxygen pressure across the BZ in swine [105].

### Numerical Methods for Tissue Model

The nonlinear equations were solved using a fully implicit finite difference scheme with a line search Newton-Raphson method. The transmembrane flux component of the Jacobian was calculated using finite differencing with the remainder calculated analytically. The Jacobian was inverted directly using MatLab and was only recalculated if the residual fails to decrease or convergence was not reached within 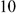 Newton-Raphson iterations.

The dependence of the BZ width on the spatial and temporal discretizations was determined to test for numerical convergence. The width of the transition for each ion concentration between the viable and ischemic region, referred to as the BZ width for each ion, was calculated by fitting a Hill curve to each ionic profile. The width of the BZ for each ion was then calculated as the distance between 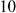 and 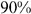 of the change in concentration.

A convergence analysis was performed and a mesh discretization of 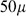
m and a time step of 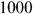
ms was used. Increasing the spatial discretization by a factor of 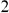 results in a maximum change in BZ width of any ion of 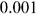
mm. Decreasing the time step to 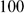
ms increases the maximum BZ width of any ion by 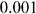
mm.

## Results

The individual effects of each of the four components of ischemia were first demonstrated. A combined model of ischemia was then developed and the width and magnitude of the changes in ionic concentrations across the ischemic BZ were predicted. The effect of movement of 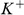, 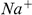 and 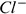 within and between intra and extracellular spaces across the BZ were calculated to show the net movement of ions across the BZ.

### Simulating Individual Components of Ischemia

**Figure 2:**
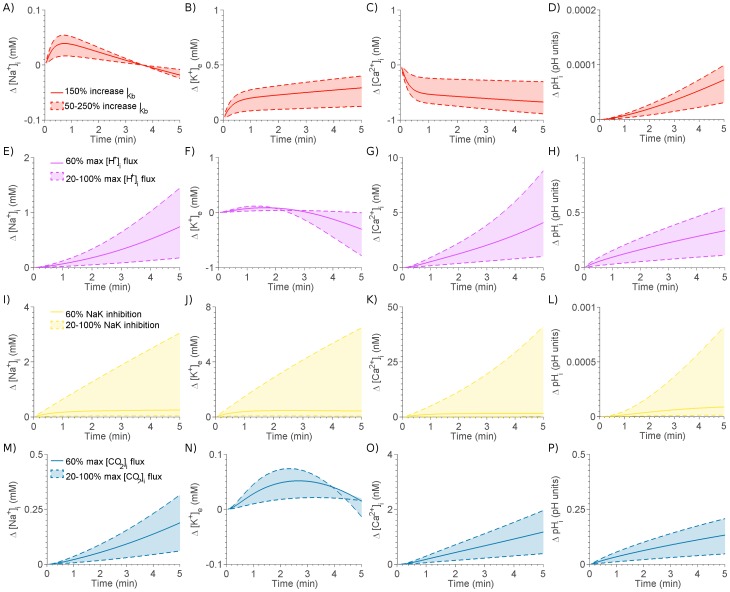
Effect of individual components of ischemia on the change in 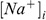 (column 1), 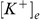 (column 2), 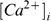 (column 3) and 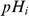 (column 4). Effect of 50–250% increase in 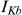 (red shaded region enclosed by dashed line) and a 150% increase in 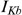 (red line) on A) 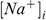, B) 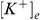, C) 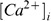 and D) 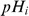. Effect of 20–100% maximum 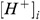 flux (purple shaded region enclosed by dashed line) and 60% maximum 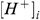 flux (purple line) on E) 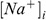, F) 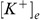, G) 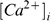 and H) 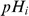. Effect of 20–100% NaK inhibition (yellow shaded region enclosed by dashed line) and a 60% NaK inhibition (yellow line) on I) 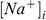, J) 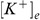, K) 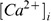 and L) 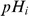. Effect of 20–100% maximum 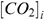 flux (blue shaded region enclosed by dashed line) and a 60% maximum 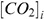 flux (blue line) on M) 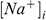, N) 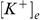, O) 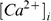 and P) 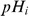.

The level of inhibition of 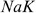, the activation of 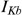 and the level of 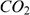 and 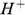 production in the cell during ischemia is not known. To investigate the effects of each of these aspects of ischemia, they were each individually introduced into the model at five levels of severity, for x
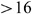
mm along a 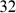
mm strand. Fig. 2 shows the effect of each component on the change in 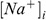, 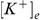, 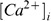 and 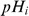 across the ischemic BZ. The range of alterations in ion concentrations is shown by the shaded regions. The minimum and maximum changes in each component are shown by dashed lines and the mid change is shown by the solid line. 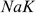 (yellow) was inhibited by up to 100% (no flux) in 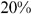 increments, 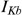 (red) was scaled by up to 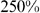 in 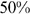 increments, metabolic acidosis was simulated by introducing an 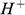 flux in five increments up to a maximum value of 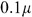
Mms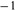 and respiratory acidosis (blue) was simulated by a intracellular 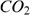 flux increased in five increments up to a maximum value of 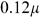
Mms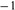.

### Modelling Ischemia

Inherently, there is no single mode of ischemia and the relative contribution of acidosis, 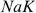 inhibition or 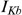 activation will depend on the residual flow, age, gender, disease state, location of ischemic region and species under study. To provide a representative case to study, 5 minutes of ischemia were simulated in the presence of all four ischemic mechanisms that match representative results from the literature.

Partial pressure measurements of 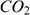 in canine ischemic models show a 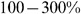 increase in 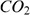 partial pressure following occlusion, depending on the level of flow inhibition, after 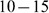 minutes. The elevation in 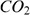 was approximately linear over time and hence in the model it was assumed 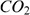 concentration increases by 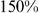 increase in the first 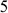 minutes of ischemia [106], [107]. This corresponded to an increase in the concentration of 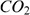 in the model from 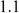
mM to 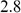
mM. In the model respiratory acidosis was caused by an increase in 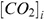 flux that cannot be vented from the extracellular space. Although it is recognised that the decrease in 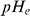 due to metabolic acidosis will also contribute to elevated 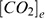, initially the 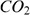 flux was set at a 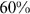 level to achieve an increase in 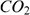 concentration to 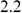
mM, which resulted in a decrease of 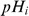 to 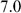. During ischemia 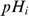 decreases rapidly before plateauing after approximately 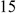 minutes. The decrease in the initial 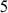 minutes has been reported to fall between 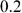 to 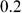 pH units in rat [108], ferret, [109] and guinea pig [110] preparations. Respiratory and metabolic acidosis will both contribute to this drop in 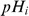. In the model setting the level of intracellular 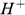 flux to 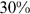 caused a 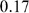 pH unit drop and an increase in 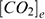 to 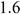
mM. Combined metabolic and respiratory acidosis caused a 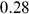 unit drop in pH and an increase in 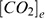 to 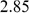
mM.

In studies of ischemia 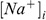 tends to increase linearly with time. In the rat heart ischemia caused an increase in 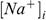 by 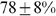 over 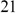 minutes [111], by 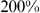 after 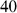 minutes [112], from 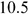
mM to 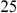
mM during 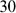 minutes ischemia [113], by 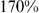, 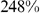, 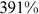 and 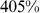 after 9, 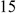, 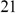 and 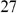 minutes, respectively [114], by two fold over 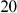 minutes [115], from 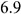
mM to 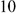
mM over 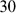 minutes [116], by 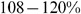 over 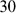 minutes [117], by 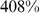 over 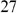 minutes [118], no change over 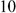 minutes [108] and by 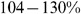 over 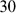 minutes [119]. Changes in 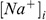 in guinea pig hearts during ischemia is controversial with reports of a decrease from 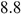
mM to 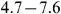
mM over 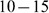
minutes [120] and of an increase of 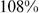 over 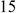 minutes [121]. Only considering the cases where 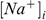 increases, as these represent a repeatable consensus result, the range of expected increases in 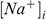 over a 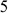 minute period is 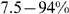, assuming a linear increase with time. These cluster into two groups with ranges 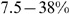 and 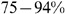. In the model reducing the maximum flux of 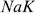 by 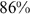 caused an increase in 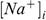 to 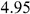
mM (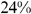) in the absence of acidosis or 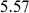
mM (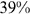) in the presence of the acidotic components of ischemia, described above.

Given the inhibition of 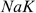 and levels of respiratory and metabolic acidosis a sweep of 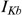 activation values was performed in the presence of these changes to achieve the desired level of extracellular 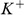 accumulation. During ischemia, 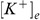 increases over three characteristic phases, with the first phase occurring during the initial 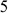 minutes of ischemia prior to reaching a plateau from minutes 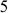 to 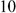, before increasing again. In guinea pigs, ischemia caused 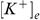 to increase from 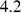 to 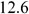
mM over 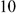 minutes [122] or 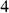 to 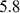
mM over 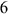 minutes [123]. In swine, ischemia caused 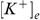 to increase from 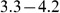
mM to 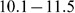
mM after 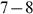 minutes [124], [125]. In rat, ischemia caused an increase from 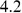
mM to 8mM over 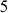 minutes [110], although other groups have seen a biphasic change in 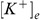 in rat with an increase from 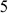
mM to 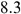
mM before falling back to 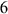
mM then continuing to rise again, observed over the first 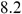 minutes [126]. In rabbit, ischemia caused 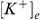 to increase from 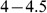
mM to 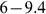
mM over 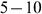 minutes [10], [110], [125]. In canine, 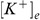 increases from 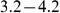
mM to 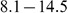
mM after 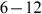 minutes [127], [128]. These results indicate an increase in 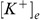 from 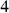
mM to 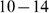
mM after 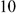 minutes and assuming 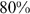 of this rise occurs in the first 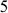 minutes [127], and combined with the 5 minute data gives an estimate of 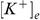 after 5 minutes of ischemia as 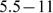
mM. In the model an increase in 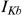 of 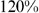 was used to achieve an elevation of 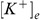 to 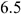
mM, resulting in an increase in the membrane potential of 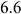
mV to 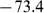
mV. This is consistent with measurements in cat (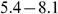
mV over 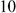 minutes) [129], [130], sheep (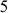
mV over 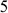 minutes) [131], guinea pigs (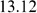
mV over 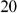 minutes) [132] and mice (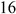
mV over 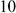 minutes) [133], but less than measurements in rabbit (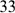
mV over 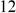 minutes [10]) and guinea pig (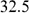
mV over 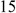 minutes [120] or 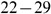
mV over 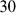 minutes [134]–[136]). The broad variation in membrane potential changes was not unexpected given the range of changes in 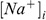 and 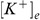 reported and in simulations an intermediate value has been achieved.

**Figure 3:**
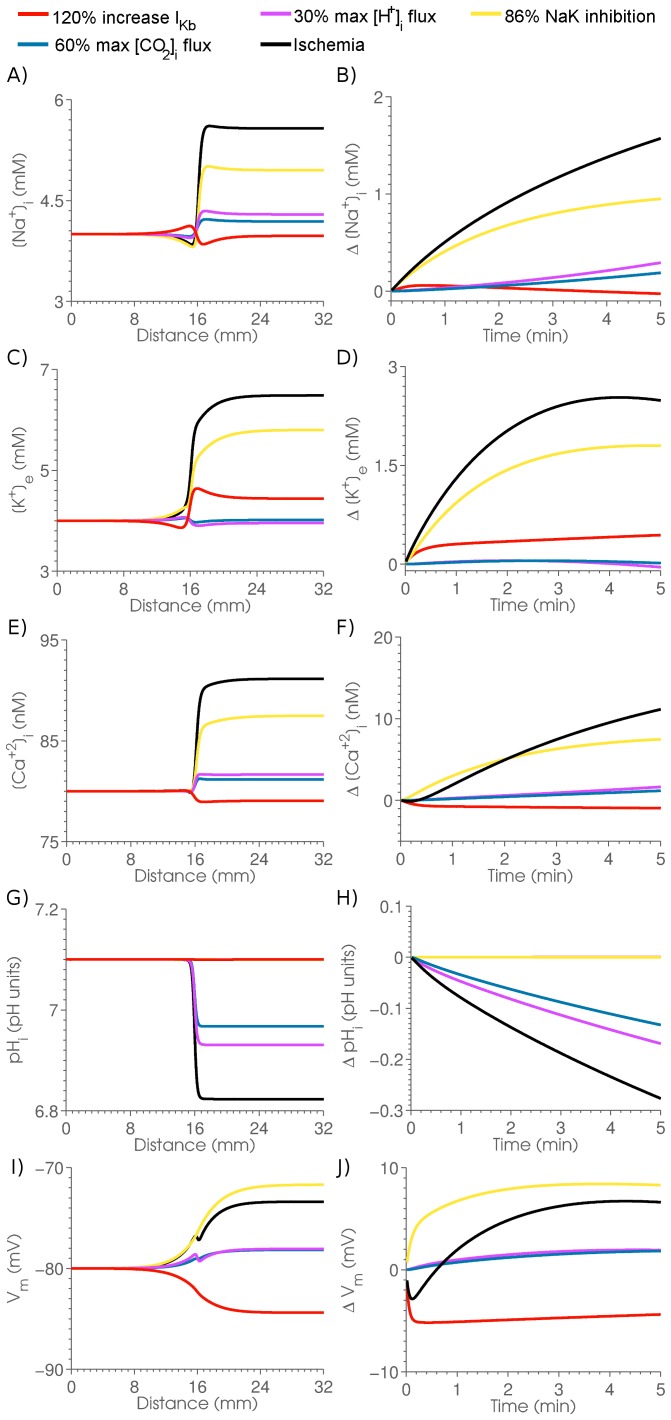
Evolution and profile of A-B) 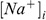, C-D) 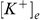, E-F) 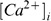, G-H) 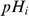 and I-J) membrane potential across the BZ due to each component of ischemia. Complete ischemia, 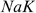 inhibition, 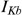 inhibition, respiratory acidosis and metabolic acidosis are represented by black, yellow, red, blue and purple lines, respectively. Column 1 shows the profile of ionic concentrations and membrane potential after 5 minutes and column 2 shows the evolution of the change in magnitude in ionic concentrations and potential across the border zone with time.

The individual and combined effects of the four components of ischemia on the temporal evolution of the maximum change in ionic concentrations and the spatial concentration and potential distribution profile after 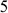 minutes of simulated ischemia are shown in Fig. 3. As ischemia progressed, 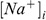 continued to increase. The early rise was attributed to 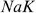 inhibition; the later increases were due to metabolic and respiratory acidosis. The early rise in 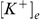 was significantly affected by 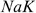 inhibition with 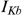 activation playing a greater role as ischemia progresses and the membrane potential diverges from the 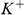 reversal potential. The decrease in 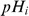 was solely due to acidosis with no impact from 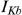 activation or 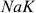 inhibition. 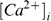 elevation was contributed to principally by 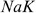 inhibition, while increased 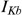 decreased 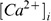. Ischemia caused an initial drop followed by a sustained rise in 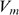. 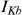 activation caused an early hyperpolarisation of the membrane potential, which was subsequently countered by the depolarising effects of 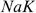 inhibition, with acidosis having a limited effect.

**Figure 4:**
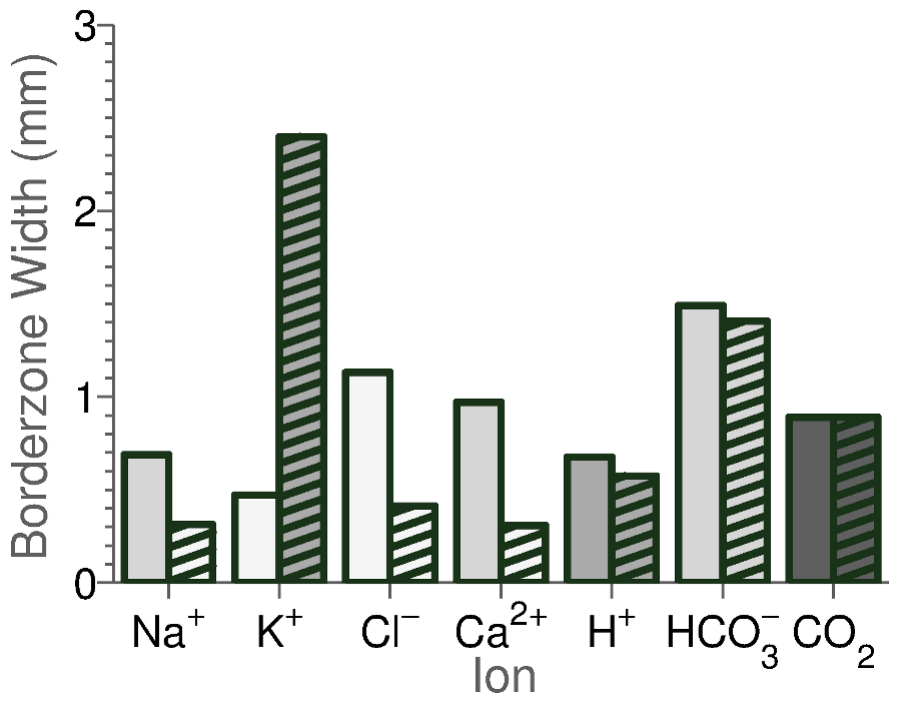
Width of ionic BZ. Gray scale represents magnitude of gradient with white indicating no gradient. Solid bars indicate intracellular gradient and striped bars indicate extracellular gradient.

Figure 4 plots the width of the BZ for each ion with striped bars corresponding to extracellular space and the darker the bar the more significant the concentration gradient relative to the initial concentration. This plot shows that 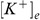 had a significantly wider BZ with greater magnitude than 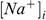. For pH regulation, 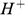 had a narrower BZ compared to the significantly wider 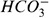 BZ, which may indicate the facilitation of proton transport via 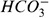 diffusion.

### Extracellular Potassium Gradients

**Figure 5:**
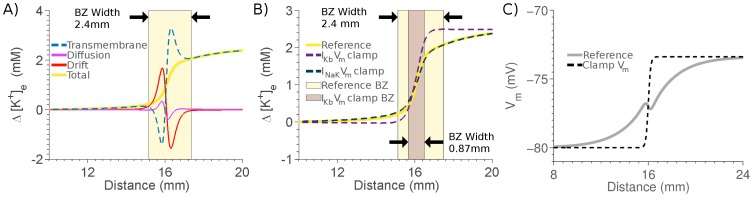
Mechanisms underpinning 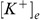
 **BZ width.** A) Reference change in 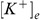 after 5 minutes of simulated ischemia caused by transmembrane flux (blue dashed line), diffusion (purple line), drift (red line) and the total change due to all causes (yellow line). The BZ is indicated by the yellow shaded region. B) Change in 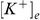 in the reference model (yellow line) compared with change in 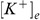 when 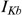 (purple dashed line) or 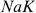 (blue dashed line) are exposed to a clamped membrane potential. The reference BZ and BZ when 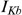 is exposed to a clamped membrane potential are indicated by the yellow and purple shaded regions, respectively.

The 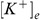 BZ width was significantly wider than other BZ ion widths and notably larger than the 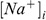 BZ width. To determine the cause of this extended 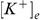 BZ the source of the cumulative changes in 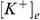 due to transmembrane flux, drift or diffusion over the 5 minutes of ischemia were calculated and plotted in Fig. 5. This showed that only the transmembrane flux had a significant gradient across the ischemic region. Separating the transmembrane flux into the 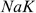 and 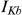 components then identified the 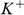 channel, which includes the ATP-inactivated 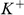 current, as the cause of this gradient. The gradient of *I_Kb_* was due to the extensive membrane potential gradient into the ischemic region (see Fig. 3). To confirm that this was the cause of the 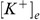 BZ width, the membrane potential was calculated as normal, but an additional clamped membrane potential was calculated at each time step. The clamped membrane potential had the same maximum and minimum values as the correct membrane potential but instead of a smooth gradient across the BZ it had a sharp transition over the BZ. A comparison of this clamped and the control membrane potentials is shown in the Fig. 5C. The effects of using a clamped membrane potential to calculate the 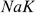 flux or the 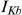 current on the cumulative changes in 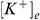 after 5 minutes of simulated ischemia are plotted in Fig. 5B, demonstrating that by removing the gradient in the membrane potential experienced by 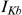 there is a significant narrowing in the 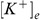 BZ.

### Drift and Diffusion

**Figure 6:**
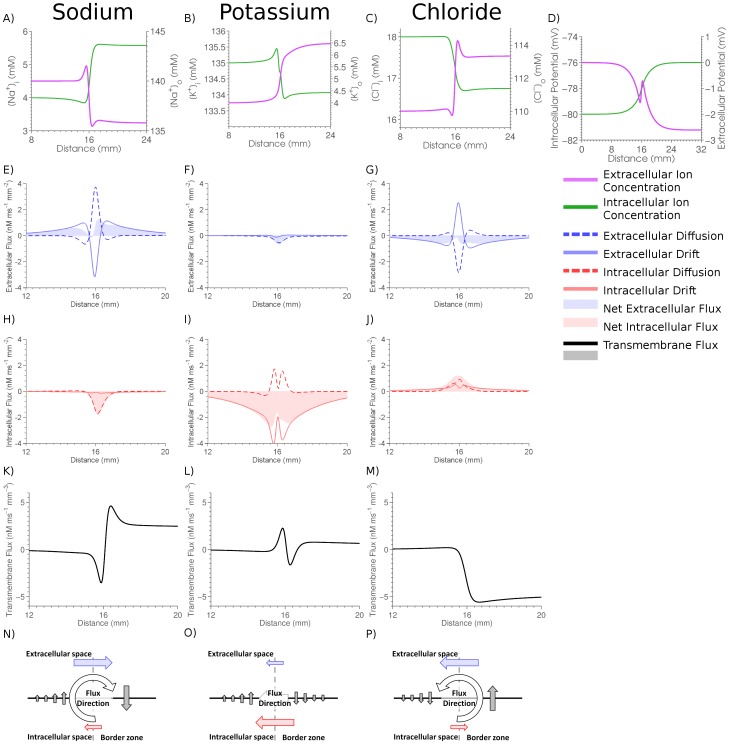
Regulation of 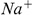, 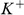 and 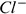 ionic concentrations across the border zone after 5 minutes of ischemia. Intra and extracellular A) 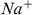, B) 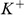 and C) 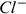 ionic concentrations. D) Intra and extracellular potential. Intracellular drift and diffusion flux of E) 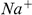, F) 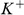 and G) 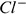. Extracellular drift and diffusion of H) 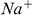, I) 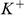 and J) 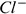. Transmembrane flux of K) 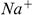, L) 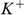 and M) 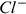. Schematics showing the general direction of ion movement within and between the intra and extracellular space for N) 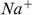, O) 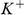 and P) 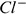.

To investigate the relative contribution of drift and diffusion to intra region fluxes (inter or extracellular) the drift and diffusion fluxes in each region were plotted, alongside the transmembrane flux, over the length of the strand after 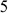 minutes of ischemia. Fig. 6 shows the differences in drift and diffusion between 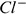, 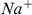 and 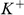. As expected from the intra and extracellular gradients, intracellular 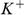 and 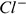 diffused into and out of the ischemic region in the intra and extracellular space, respectively. The converse was the case for 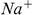. Due to the decrease in transmembrane potential, characteristic of ischemic regions, there was a convergence of intra and extracellular potentials with the extracellular potential decreasing in the ischemic region and the intracellular potential increasing. This gradient caused positive ions to drift into the ischemic region in the extracellular space and drift out of the ischemic region in the intracellular space. The converse was true for negatively charged ions. As drift is proportional to the ionic concentration, 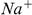 drift was significant in the extracellular space and 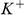 drift was significant in the intracellular space. For 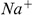, drift and diffusion operated in the same direction in the intra and extracellular space. The result was a cyclical movement of 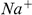 moving into the ischemic region in the extracellular space, while moving out of the ischemic region in the intracellular space. The 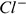 movement was also circular but in the opposite direction (Fig. 6P). However, for 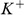 drift and diffusion were in opposite directions. In the extracellular space where the 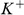 concentration was low, diffusion dominated and 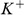 moved out of the ischemic region. In the intracellular space, where there was a higher concentration of 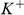, drift dominated, also causing 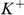 ions to move out of the ischemic region. Thus ischemia caused a depletion of 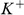 in the ischemic region through both the intra and extracellular space and, contrary to previous hypothesis [13], the model suggests that intracellular 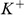 movement is the dominant path for 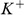 to leave the ischemic region.

## Discussion

In this study a new model of cardiac tissue electrophysiology was developed. The model predicted that the width of the 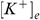 gradient across an ischemic BZ would be significantly wider the 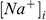 BZ. The cause of this difference was attributed to the voltage dependence of the 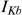 channel. The model also demonstrated that, due to electrogenic drift, 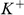 moved out of the ischemic region in both the intra and extracellular space which will lead to 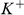 depletion.

The model of ionic movement and tissue electrophysiology was developed by combining the Nernst-Plank equations with the bidomain framework. No attempt was made to explicitly validate the proposed tissue model equations due to the paucity of experimental data. However, applying simplifying assumptions with regards to ionic or voltage gradients reduces the proposed equations to the well validated bidomain equations [137], [138] or coupled reaction-diffusion equations [28], [86], respectively, providing support for the validity of the proposed modelling framework. The limited attempts at simulating the spatial temporal evolution of ionic gradients across the ischemic BZ have largely uncoupled the movement of ions and the electric field. Potse et al., [18] demonstrated that measured 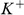 gradients across an ischemic BZ could be simulated using a model of 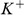 diffusion coupled to a source term. The spatially varying 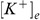 gradient could then be included as a boundary condition to models of transmembrane current in the bidomain equations. This model did not include any effect of electric gradients on 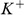 movement, 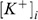 movement, inter ionic species interactions or the effect of the ischemic region on any other ion gradient. Similar sets of equations to those proposed here have been used for simulating the potential gradient surrounding cells, including the Debye layer [139], electrical propagation along strands of cardiac cells [140], [141] and for modelling ion diffusion in the cable equation [142]. These previous models have either explicitly represented the intra and extracellular domains or only considered the intracellular domain but have not modelled the tissue within the bidomain framework, as derived and implemented here.

The proposed equations can be applied generally in three dimensions as opposed to the one dimensional simulations presented here. The current study does not consider the effects of anisotropy on ion or membrane potential gradients across the BZ, however, if implemented in two or three dimensions the model is capable of representing tissue anisotropy and any effects this may have on BZ gradients. By conserving ionic species the proposed equations provide a more biophysical representation of cardiac electrophysiology than the bidomain equations and can appropriately be applied to simulate a broader range of conditions. However, these benefits come at a cost. Unlike the bidomain equations, with two partial differential equations that can readily be uncoupled and solved as two sets of linear equations [143], the proposed model is nonlinear and contains two parabolic partial differential equations for each ionic species and one elliptic partial differential equation to model the electric potential. This results in a significant increase both in the complexity and number of equations that must be solved and hence comes at a significant increase in computational cost. The proposed framework can be used to simulate electrically active tissue by introducing a full action potential cell model [31]. This would require identifying and separating out the transmembrane pathways for each ionic species present in the cell model to calculate the net transmebrane flux for each ion or 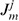 in Eqn 18. The internal cell model state variables, including gating variables, Markov states and intracellular 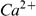 dynamics would also need to be solved, introducing a system of nonlinear ordinary differential equations at each grid point. The small time steps and increased number of degrees of freedom required to simulate electrically active tissue would further increase the computational cost of the proposed model.

A new model of ion movement across the un-stimulated cell membrane was developed. This model has been published online and is available at cellml.org. The model creation approach demonstrated two novel methods. Firstly, the ion transporter densities were uniquely constrained by a small number of experimental data sets and a zero net flux constraint. This provided a repeatable and unique method for determining model parameters under quiescent conditions and could readily be applied to any cardiac electrophysiology model to constrain model parameters. Secondly, in the development of this model a grid of 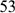 experimental observations were created to provide a comprehensive validation of the model response to changes in ion concentrations and in the presence of blockers of major transporters. Although these observations only provide a qualitative comparison, they do provide a benchmark for quantifying the generality of models. Furthermore, Table 4 identified 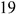 experiments that do not appear to have been performed or remain controversial, highlighting the potential for additional experiments.

In this study the process of validating the model against 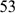 experimental observations demonstrated the general capacity of the model to replicate the majority of experimental results. However, the model was unable to match ten of the 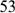 observations. Five of the experimental observations found no change in a measurement. Due to the numerical nature of the model, even very small changes in a concentration can be observed and without adding in a semi arbitrary threshold that should reflect the variability and confidence of each experimental measurement it was not possible for the model to return no change in a value. In general the five remaining failed observations can be attributed to absent mechanisms in the model or inconsistencies with the model and specific experimental setups. The model did not predict the 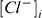 response to depressed 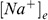 and the 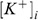 response to depressed 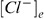. This is potentially due to the absence of NaK2Cl from the model, largely due to the lack of data characterising the transporter. The model was unable to replicate the decrease in 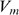 due to elevated 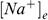. This may be due to the specifics of the experimental protocol. Decreasing 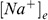, which should have the opposite effect on 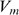, caused both increases and decreases in 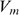 in different studies indicating that the 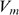 dependence on 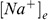 is sensitive to the specifics of the experimental setup. The model did not predict the change in 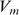 or 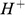 with a decrease in 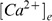. Experimental observations report an increase in 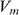 with both a decrease or an increase in 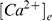. This may indicate that the 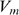 is at some minima with respect to 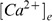, although this seems unlikely. It more likely reflects inconsistent data due to differences in experimental setups that were impossible for the model to replicate. In cardiac myocytes it is postulated that 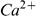 and 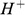 compete for common buffering sites [144], [145]. The decrease in 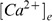 may drain the cell of 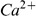, reducing the 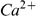 bound to buffers that could then be occupied by 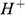, resulting in a decrease in 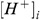. A common pool of 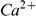 and 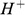 buffering was not present in the model and this may explain the disparity between model predictions and experimental results. Despite the inability of the model to replicate 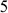 of the 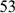 observations, the majority of the absent mechanisms are expected to play a secondary role during ischemia. NaK2Cl is a potential contributor to the elevation in 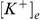 [146], although this has been questioned [131], and its absence is unlikely to affect the general model conclusions.

Models simulations found a BZ width for the different ions of between 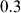
mm (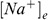) and 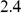
mm (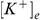). The variation in the width of each ion across the BZ means that there was no single BZ width predicted by the model. The majority of ions transitioned from viable to ischemic concentrations over 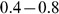
mm, with 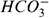 and 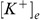 notable outliers. The model predictions are consistent with previous measurements of the BZ between 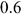 and 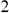
mm [10], [11], [147]–[149]. However, the model did not include the sharp gradients in metabolites over 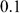
mm [11] or the effects of regions of reduced perfusion affecting mechanics over 20 mm distance from the ischemic region [150].

The model predicted that 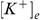 and 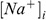 have significantly different BZ widths (Fig. 4). It was expected that the coupling of 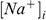 and 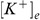 via 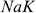 would result in concordant gradients in these two ions across the BZ. The extended 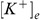 gradient was attributed to the voltage dependence of the 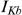 (Fig 5). Unlike 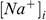, 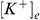 is regulated by two electrogenic transmembrane pathways 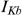 and 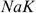, whereas 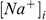 is regulated by multiple exchangers that are electro neutral, or have attenuated voltage dependence, compared to ion channels. This discordance in 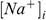 and 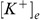 gradients will have a significant effect on the gradient of action potential morphology across the BZ. Elevation of 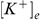 depolarises the cell and shortens the action potential duration, whereas elevation of 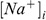 causes a reduction in action potential duration [151], [152]. The combined effect of the two gradients will be a slower change in resting membrane potential on the length scale of the 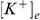 gradient and a much more rapid transition in action potential duration due to the faster change in 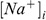 in conjunction with the change in 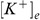 over the BZ. The temporal evolution of the two gradients are also distinct, with a sustained constant increase in 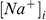 over the first 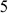 minutes, while 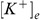 increases rapidly for 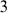 minutes before plateauing (Fig. 3). These simulation results are consistent with experimental measurements of ionic concentrations [111], [152] and changes in ECG morphology, which report an early elevation of resting membrane potential, followed by a decrease in action potential duration [153] during early ischemia. These spatial-temporal increases in ion concentrations and secondary effects on electrophysiology will increase tissue heterogeneity and have the capacity to play an important role in the BZ arrhythmogenic substrate.

Experimental measurements have reported a decrease in 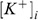 during ischemia [152]. Loss of 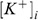 has been attributed to a combination of increased 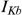 conduction and 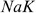 inhibition [152], [154]. However, depletion of 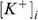 due to transmembrane 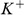 movement, in the absence of a potential gradient, would cause an intracellular flux of 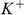 into the ischemic zone, due to diffusion, to replenish 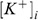, mitigating the effects of changes of 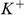 transmembrane flux on 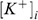. The model proposed here demonstrated that this is not necessarily the case. The model predicted a significant electrogenic 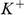 flux in the intracellular space out of the ischemic zone (Fig. 6), resulting in a net efflux of 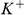 out of the ischemic zone both in the intra and extracellular space. The movement of 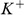 in the intracellular space out of the ischemic zone would further deplete 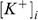 in the ischemic region and exacerbate 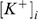 loss, but may limit 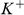 accumulation in the extracellular space.

### Limitations

The model is inherently an approximation and hence represents a finite set of known cellular properties and changes that occur during ischemia. In particular, the model treated all buffers as static and rapid, the effects of protons on channel, exchanger and co-transports were not considered, ischemic changes were instantaneous and the model did not include all possible changes or pathways that may affect ionic homeostasis during ischemia.

The tissue model assumed that 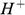 and 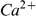 buffers are static, rapid and made up of a single population of binding sites. It is known that some of the buffers for both 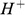 and 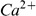 are mobile, these could be introduced into the model framework as an additional concentration but these effects were approximated, without the additional computational cost of adding a additional ionic concentration, by using effective diffusion constants. The equilibrium assumption was likely to be valid in the current model due to the long time scales of interest; however, simulation of cardiac action potentials would require the re-evaluation of this assumption. It is also known that 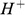 [28] and 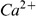 [155] are buffered by multiple proteins with distinct binding kinetics but over the range of concentrations simulated these multiple buffer species were unlikely to have significant effects.

The model of intracellular 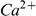 dynamics assumes that the SR and cytosolic 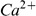 concentrations remain in equilibrium, which is clearly not the case during an action potential. The model of intracellular 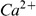 dynamics provided a numerically efficient representation of intracellular 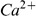 buffering and SR 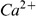 uptake. The use of a Hill coefficient of one for SERCA as opposed to the more common and biophysical value of two removed the need for an additional differential equation to model 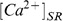 or the solution of a set of nonlinear equations to model 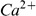 regulation. Furthermore, over the range of 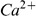 values studied it was possible to adjust the maximum SERCA flux to minimize discrepancies between a model with a Hill coefficient of one or two.

It is well recognised that 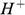 play an important role in regulating cellular electrophysiology [156]. Experimental and modelling studies have demonstrated the effects of 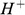 on ryanodine receptor opening probabilities, 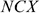, 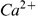 channels, 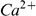 buffering and SERCA [22], [157]. The majority of the effects of 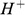 on 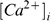 regulation are unlikely to play a significant role in determining the spatial and temporal 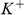 and 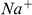. However, inhibition of 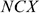 potentially contributes to 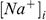 gradients but this will be secondary to the effects of 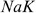 inhibition.

The model treats all changes for ischemia as instantaneous. The time dependence of inhibition of 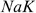, activation of 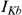, increased 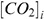 flux or increased 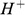 flux are not known and can only be approximated. A linear ramp in ischemic changes has been used previously, but this fails to consider the possibility that changes occur over different time scales. In order to minimise ambiguity in model simulations an instantaneous change in 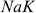, 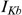, 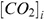 flux and 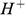 flux was chosen.

To limit the scope of this study, the effects of cell swelling and the effects of this on changes in ion concentrations [122] were not included in the model. However, previous, modelling studies have found these effects to not significantly alter 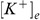 accumulation and may not fundamentally alter the study conclusions [20]. Mitani and Shattock [146] identified the Na dependent potassium channel as a potential contributor to the elevation of 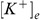. However, the effects of an increase in any 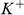 current are captured by the elevation in 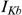 that does not necessarily need to be the sole result of an increase in conduction in the ATP inactivated 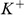 channel. Furthermore, the model does not include an increased 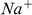 channel conductance during ischemia. This has been reported during ischemia in the form of increased permeability of 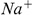 through the persistent 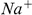 current [116] and the ATP activated 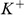 channel [158]. However, other groups have found a limited impact of the persistent 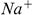 channel in 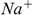 accumulation during ischemia [159] and previous modelling studies have shown that its inclusion is not required to capture the salient features of ischemia in the single cell [20]. For these reasons a potential increase in the 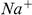 channel conductance was not included in this study.

### Summary

A new mathematical framework was derived for simulating cardiac tissue electrophysiology with ion species conservation. The model was used to simulate the movement of ions due to transmembrane channels, pumps and transporters, diffusion and drift in the intra and extracellular space. The model predicted that 1) the sodium BZ is approximately a quarter of the length of the potassium BZ and this is due to the effects of the membrane potential gradient on 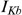 and 2) that during ischemia there is a gross movement of potassium ions out of the ischemic region in both the intra and extracellular space due to the effects of drift, which will lead to a depletion of 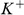 from the ischemic region.
